# Prevalence and risk of colorectal polyps among the Korean population under 50 years

**DOI:** 10.1097/MD.0000000000029493

**Published:** 2022-07-08

**Authors:** Su Jin Jeong, Jinho Lee, Eunju Kim, Jun Seong Hwang, Jin Lee, Joon Hyuk Choi, Nae-Yun Heo, Jongha Park, Seung Ha Park, Tae Oh Kim, Yong Eun Park

**Affiliations:** a Division of Gastroenterology, Department of Internal Medicine, Inje University College of Medicine, Haeundae Paik Hospital, Busan, Republic of Korea.

**Keywords:** colorectal adenoma, colorectal neoplasia, prevalence, risk factor, young adults

## Abstract

Colorectal cancer is a common cancer; generally, adults aged ≥ 50 years are screened using stool occult blood tests and colonoscopy. However, colorectal adenoma and cancer have been found in patients under the aged of 50, and studies on characteristics and risk factors in young patients are lacking. We evaluated the prevalence and risk factors of colorectal adenoma and cancer in young adults aged under 50 years.

We retrospectively analyzed 570 individuals aged under 50 years who underwent colonoscopy at the Haeundae Paik Hospital, Korea, from January to June 2018. Logistic regression model was used to identify the risk factors for colorectal adenoma and colorectal cancer.

The prevalence of colorectal adenoma in group of 19–29 years was 3.2% (1 of 31), 30–39 years was 13.8% (30 of 217) and in the group of 40–49 years was 21.1% (68 of 322) (*P* = .009). In multivariable analysis, age over 45 years (adjusted odds ratio [OR], 1.941; 95% confidence interval [CI], 1.187–3.172; *P* = .008) and male sex (adjusted OR, 1.711; 95% CI, 1.044–2.806; *P* = .033) were independent risk factors for colorectal neoplasia including cancer.

The prevalence of colorectal adenoma increases as the age increased in young adults under 50 years of age, especially after the age of 45 years, the risk of colorectal neoplasia increases; hence, early screening should be considered before the age of 50 years.

## 1. Introduction

Colorectal cancer (CRC) is a malignant tumor that occurs in the colon and rectum. According to a recent survey by Bray et al,^[1]^ it was the third most common carcinoma worldwide and the second most common cause of cancer death in 2018. In Korea, CRC was the second most common cancer in 2017 and the third most common cause of cancer death in 2018.^[2]^ Genetic and environmental factors affect CRC, and risk factors for CRC include age over 50 years, dietary factors (high fat and meat intake and low dietary fiber intake), alcohol consumption, obesity, and family history of CRC.^[3,4]^ Colonoscopy is the most powerful modality for detecting colorectal polyps and CRC. The current international guidelines recommend screening using colonoscopy from the age of 50 years for early detection of CRC and colorectal adenomatous polyps.^[5]^ Most CRCs occur in adenomas through the traditional adenoma–carcinoma sequence.^[6]^ Screening colonoscopy for early detection of colon polyps and polyps removal can reduce the prevalence and mortality of CRC.^[7–9]^ Over recent decades, the prevalence and mortality of CRC in individuals aged ≥50 years have decreased, but those in individuals aged <50 years has increased.^[10,11]^ According to the recent trend, Bailey et al.^[10]^ predicted that in 2030, the prevalence of colon and rectal cancer will increase by 90% and 124% in individuals aged 20 to 34 years, respectively, and by 28% and 46% for individuals aged 35 to 49 years, respectively. Previous studies have reported that early-onset CRC in young adults aged under 50 years has a more advanced disease course at the time of diagnosis than that in adults aged >50 and the that prognosis is often poor.^[12,13]^ Thus, young-onset colorectal neoplasia, including CRC and its precursors (colorectal adenoma), are of great concern. However, limited studies have assessed the prevalence and epidemiology of CRC and colorectal adenoma in younger populations.

In Korea, the clinical characteristics, related risk factors, and prevalence of early-onset CRC and colorectal adenoma in patients aged <50 years have been studied in several reports, but there have been limited related studies, and most studies have been limited to specific age groups (e.g., 20–39, 40–49, or 40–59 years).^[14–17]^ Therefore, this study aimed to investigate the prevalence, clinical characteristics, and risk factors of early-onset colorectal adenoma and CRC in young adults aged 19 to 49 years.

## 2. Methods

### 2.1. Study population

**Figure. 1.**
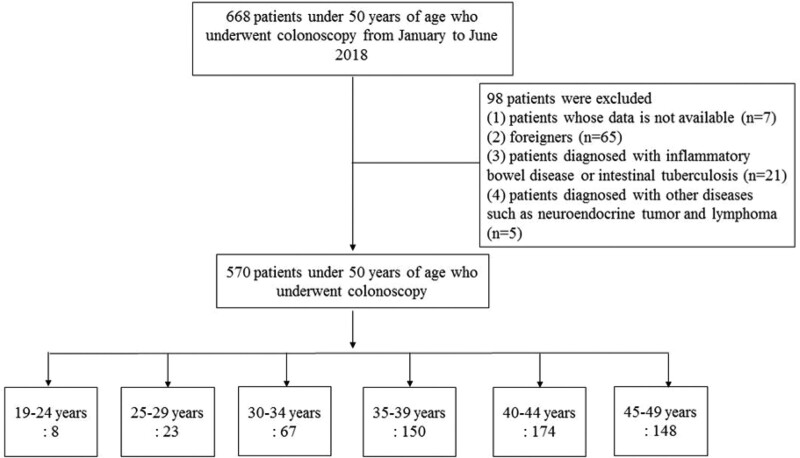
Subjects enrollment.

This study enrolled 668 patients aged <50 years who underwent colonoscopy between January and June 2018 at Haeundae Paik Hospital, Inje University College of Medicine, Busan, Korea. Patients with no clinical data or clinical records available due to incomplete examination or poor bowel preparation (n = 7), foreigners (n = 65), patients diagnosed with inflammatory bowel disease (e.g., ulcerative colitis or Crohn disease) or intestinal tuberculosis prior to colonoscopy (n = 21), and patients diagnosed with other diseases such as neuroendocrine tumor and lymphoma on colonoscopy (n = 5) were excluded. Also, polyposis caused by genetic factors such as familial polyposis was excluded. Poor bowel preparation was defined as “presence of large amounts of solid fecal matter, precluding a satisfactory study; unacceptable preparation; <90% visibility of the mucosa.”^[18]^ Finally, 570 patients were included in the study (Fig. 1). This study was performed in accordance with the ethical guidelines of the Declaration of Helsinki 1975 and approved by the Institutional Review Board of Haeundae Paik Hospital (HPIRB File No: 2020-07-017). The requirement for informed consent was waived because only de-identified data were retrospectively assessed.

We analyzed baseline characteristics of subjects according to the age. The analysis was first divided into three groups: <30 (19–29 years), 30 to 39, and 40 to 49 years. Second, the analysis, as well as colorectal adenoma and CRC risk factors, was divided into 2 groups: those aged <45 years and those aged ≥45 years.

### 2.2. Study procedures and definitions.

#### 2.2.1. Colonoscopy.

All colonoscopies were performed by experienced board-certified endoscopists using standard colonoscopes (Evis Lucera CV-260SL or Evis Lucera CV-290; Olympus Medical Systems, Tokyo, Japan). Bowel preparation was performed using a standard protocol with polyethylene glycol lavage or sodium phosphate. The polyp location, size, number, and morphology were recorded during colonoscopy. The polyp location was categorized as the right colon, left colon, and both sides. The right colon was defined as the cecum, ascending colon, and transverse colon, and the left colon was defined as the descending colon, sigmoid colon, and rectum. The size of each polyp was estimated using open 8-mm biopsy forceps (FB-230U; Olympus Medical Systems, Tokyo, Japan).^[19]^ The polyp morphology was described according to the Paris classification.^[20]^ All detected polyps were removed endoscopically using either cold forceps biopsy or endoscopic mucosal resection/endoscopic submucosal dissection.

#### 2.2.2. Diagnosis of colorectal polyps.

All detected polyps were histopathologically evaluated according to the World Health Organization criteria.^[21]^ In the histopathological examination of polyp lesions, hyperplastic polyps, chronic nonspecific inflammation, and benign lymphocytic hyperplasia were defined as normal groups. In the adenoma group, tubular adenomas measuring ≥10 mm in diameter, villous adenomas, adenomas with ≥25% change in the villous structure, tubulovillous adenomas, sessile serrated adenomas, and adenomas with high-grade dysplasia were defined as advanced adenomas. Adenomatous polyps were defined as tubular adenoma <10 mm and advanced adenoma group. CRC was defined as histopathologically confirmed adenocarcinoma. Colorectal neoplasia was defined as adenomatous polyps and CRC, and advanced neoplasia was defined as the advanced adenoma group and CRC.

### 2.3. Measurements and risk factor assessment

To investigate the medical history and drugs used in combination, endoscopists asked questions or administered self-administered questionnaires were used prior to colonoscopy. During colonoscopy, the presence of symptoms related to colon and other comorbid diseases (e.g., diabetes, hypertension, dyslipidemia, liver diseases, hematologic diseases, cardiologic diseases, and other cancers), surgical history, and drug use were examined. Body mass index was calculated as weight (in kilograms) divided by height (in meters) squared. Blood samples for biochemical and metabolic parameters were obtained after overnight fasting for at least 8 hours. Fasting plasma glucose, total cholesterol, low-density lipoprotein cholesterol, triglyceride, and high-density lipoprotein cholesterol levels were measured. Patients who were taking antihypertensive drugs or had increased blood pressure on the day of the test (blood pressure ≥130/85 mm Hg) were considered as having hypertension, and those with elevated triglyceride levels (≥150 mg/dL) and low-density lipoprotein cholesterol levels (≥130 mg/dL) were considered as having dyslipidemia. Participants diagnosed with CRC underwent additional investigations to assess tumor markers and carcinoembryonic antigen (CEA) levels. In participants who also underwent esophagogastroduodenoscopy on the day of colonoscopy, the presence of *Helicobacter pylori* infection was evaluated. *H. pylori* infection was diagnosed by histological identification using a modified Giemsa stain or rapid urease test during gastroscopy.

### 2.4. Statistical analysis

Variables are presented as mean ± standard deviation or n (%). The baseline characteristics were compared using independent Student t-test or ANOVA test, and the Mann–Whitney test for continuous variables and the χ^2^ test or Fisher exact test for categorical variables, as appropriate. Basic characteristics were compared according to age ≥45 years and <45 years and 10-year age groups. Independent predictors of colorectal adenoma and CRC in patients who underwent colonoscopy were analyzed using logistic regression analysis. Odds ratios (ORs) and the corresponding 95% confidence intervals (CIs) were calculated. Data analysis was performed using SPSS (version 25.0; IBM Corp., Armonk, NY). Furthermore, we expressed the difference between the pathological results of polyps according to 5-year age groups by group graphs using GraphPad Prism 6.0 (GraphPad Software, La Jolla, CA). A *P* value <.05 was considered statistically significant.

## 3. Results

### 3.1. Baseline characteristics and polyps of study participants

#### 3.1.1. 19 to 29 versus 30 to 39 versus 40 to 49 years.

From January to June 2018, 570 patients aged <50 years who underwent colonoscopy were analyzed. There were 31 (5.4%) patients aged 19 to 29 years, 217 (38.1%) patients aged 30 to 39 years, and 322 (56.5%) patients aged 40 to 49 years. The baseline characteristics of the included patients are shown in Table 1. Younger patients (19–29 years) were more likely to undergo colonoscopy due to symptoms than colonoscopy for either screening, other cancer work up or previous polyp (71.0% vs 19.4% vs 25.8%; *P* = .015). Patients aged ≥30 years had more underlying diseases such as hypertension (0% vs 0.9% vs 4.3%; *P* = .038), liver disease especially fatty liver (0% vs 23.5% vs 20.8%; *P* = .001), and dyslipidemia (9.7% vs 38.7% vs 39.8%; *P* = .004) than patients aged <30 years (Table 1).

**Table 1:** Baseline characteristics of study subjects by age (3 groups).

Variables	19 to 29 y (n = 31, 5.4%)	30 to 39 y (n = 217, 38.1%)	40 to 49 y (n = 322, 56.5%)	*P* value*
Male sex	15 (48.4)	120 (55.3)	154 (47.8)	.227
Median age (y)	26.2 ± 3.1	35.8 ± 2.6	44.2 ± 2.9	<.001
BMI (kg/m^2^)	21.9 ± 3.2	24.1 ± 4.2	23.5 ± 3.6	.560
Fasting glucose (mg/dL)	87.3 ± 7.8	90.6 ± 17.3	93.9 ± 23.1	.230
Cause of colonoscopy				<.001
Symptoms	22 (71.0)	42 (19.4)	83 (25.8)	.015
Hematochezia, anemia	12 (54.5)	7 (16.7)	17 (20.5)	
Dyspepsia, abdominal pain	3 (13.6)	18 (42.9)	35 (42.2)	
Bowel habit changes	7 (31.8)	14 (32.3)	28 (33.7)	
Weight loss	0 (0)	3 (7.1)	3 (3.6)	
Other cancer work up or previous polyp	1 (3.2)	7 (3.2)	15 (4.7)	
Screening	8 (25.8)	168 (77.4)	224 (69.6)	
Underlying disease				
Hypertension	0 (0)	2 (0.9)	14 (4.3)	.038
Diabetes	0 (0)	3 (1.4)	15 (4.7)	.061
Liver disease				.001
Fatty liver	0 (0)	51 (23.5)	67 (20.8)	
Viral hepatitis	0 (0)	1 (0.5)	9 (2.8)	
Others†	2 (6.5)	0 (0)	4 (1.2)	
Cardiologic disease	1 (3.2)	0 (0)	4 (1.2)	.112
Other malignancy	0 (0)	5 (2.3)	19 (5.9)	.061
Dyslipidemia	3 (9.7)	84 (38.7)	128 (39.8)	.004
*Helicobacter pylori* infection (n = 90)‡	2 (33.3)	10 (40.0)	33 (55.9)	.287

Data are expressed as mean ± standard deviation (SD) or n (%).

BMI = body mass index.

* *P* value for comparing patients with 3 groups divided by age.

† Others: alcoholic hepatitis, Wilson disease, toxic hepatitis, cryptogenic LC.

‡ A total of 90 subjects were tested for *Helicobacter pylori* infection. A total of 45 subjects were identified as positive for *Helicobacter pylori* infection.

The polyp detection rate increased with the increasing age (9.7% vs 35.9% vs 40.7%, 19–29, 30–39, 40–49, respectively; *P* = .003; *P* = .003), and the ratio of tubular adenomas < 10 mm with low-grade dysplasia was higher (3.2% vs 13.8% vs 21.1%; *P* = .009). The prevalence of advanced adenomas increased with the increasing age (0% vs 0.5% vs 1.6%; *P* = .400), and the location of advanced adenomas tended to be higher in the right colon than in other locations. CRC was only found in patients aged ≥40 years (0% vs 0% vs 1.6%; *P* = .143; Table 2). One out of 5 patients with CRC was diagnosed with CRC at our hospital, and additional evaluation and treatment were performed at other hospital, so the final stage could not be confirmed. Of the remaining 4, 1 was identified as stage IIIA and 3 as stage IV. The CEA values of patients diagnosed with CRC were 2.52 ± 1.34 (ng/mL), which was within the normal range for most of them (data not shown).

**Table 2:** Characteristics of polyps in 3 groups of subjects divided by age.

Variables	19 to 29 y (n = 31, 5.4%)	30 to 39 y (n = 217, 38.1%)	40 to 49 y (n = 322, 56.5%)	*P* value*
Polyps	3 (9.7)	78 (35.9)	131 (40.7)	.003
Location				.062
Right colon	0 (0)	34 (43.6)	57 (43.5)	
Left colon	3 (100.0)	32 (41.0)	41 (31.3)	
Both	0 (0)	12 (15.4)	33 (25.2)	
Morphology				.096
Is	2 (66.7)	73 (93.6)	112 (85.5)	
Ip	0 (0)	2 (2.6)	5 (3.8)	
Isp	1 (33.3)	1 (1.3)	6 (4.6)	
IIa	0 (0)	2 (2.6)	8 (6.1)	
Size (mm)	9.67 ± 9.07	4.27 ± 2.43	5.84 ± 8.33	.234
Number (n)	0.11 ± 0.32	0.76 ± 1.03	0.95 ± 1.43	.659
Pathologic findings				
CNSI	0 (0)	15 (6.9)	32 (9.9)	.105
HP	2 (6.5)	47 (21.7)	64 (20.1)	.140
TA, LGD < 10 mm	1 (3.2)	30 (13.8)	68 (21.1)	.009
TA, LGD ≥ 10 mm	0 (0)	1 (0.5)	1 (0.3)	.905
TVA/VA	0 (0)	0 (0)	1 (0.3)	.680
SSA	0 (0)	0 (0)	3 (0.9)	.313
TA, HGD	0 (0)	0 (0)	1 (0.3)	.680
Adenomatous polyps	1 (3.2)	30 (13.8)	69 (21.4)	.007
Advanced adenomas	0 (0)	1 (0.5)	5 (1.6)†	.400
Location				.301
Right colon	0 (0)	0 (0.0)	3 (60.0)	
Left colon	0 (0)	1 (100.0)	1 (20.0)	
Both	0 (0)	0 (0.0)	1 (20.0)	
Colon cancer	0 (0)	0 (0)	5 (1.6)	.143

Data are expressed as mean±standard deviation (SD) or n (%).

CNSI = chronic nonspecific inflammation, HGD = high-grade dysplasia, HP = hyperplastic polyp, LGD = low-grade dysplasia, SSA = sessile serrated adenoma, TA = tubular adenoma, TVA = tubulovillous adenoma, VA = villous adenoma.

* *P* value for comparing patients with three groups divided by age.

† Advanced adenomas were found in 5 subjects, in one subject, one TVA and one TA, HGD were detected respectively. A total of 6 advanced adenomas were detected in the age of 40- to 49-year group.

#### 3.1.2. Under 45 vs >45 years.

There were 422 (74.0%) patients aged 19 to 44 years and 148 (26.0%) patients aged 45 to 49 years. The baseline characteristics of patients in both groups are shown in Table 3. The prevalence of diabetes (2.1% vs 6.1%; *P* = .018) and other malignancies (2.6% vs 8.8%; *P* = .001) was significantly higher in patients aged ≥45 years than in those aged <45 years. A total of 90 subjects were tested for *H. pylori* infection. There was no significant difference in *H. pylori* infection between groups, but about 50% of the 90 patients tested in both groups had *H. pylori* infection (Table 3).

**Table 3:** Baseline characteristics of study subjects by age (2 groups, 45-years-old standard)

Variables	19 to 44 y (n = 422, 74.0%)	45 to 49 y (n = 148, 26.0%)	*P* value*
Male sex	219 (51.9)	70 (47.3)	.336
Median age (y)	37.6 ± 4.9	47.0 ± 1.4	<.001
BMI (kg/m^2^)	23.8 ± 3.9	23.2 ± 3.6	.142
Fasting glucose (mg/dL)	91.5 ± 18.7	94.5 ± 25.0	.149
Cause of colonoscopy			.045
Symptoms	99 (23.5)	48 (32.4)	.402
Hematochezia, anemia	28 (28.3)	8 (16.7)	
Dyspepsia, abdominal pain	36 (36.4)	20 (41.7)	
Bowel habit changes	32 (32.3)	17 (35.4)	
Weight loss	3 (3.0)	3 (6.3)	
Other cancer work up or previous polyp	15 (3.6)	8 (5.4)	
Screening	308 (73.0)	92 (62.2)	
Underlying disease			
Hypertension	9 (2.1)	7 (4.7)	.100
Diabetes	9 (2.1)	9 (6.1)	.018
Liver disease			.076
Fatty liver	96 (22.7)	22 (14.9)	
Viral hepatitis	5 (1.2)	5 (3.4)	
Others†	4 (0.9)	2 (1.4)	
Cardiologic disease	2 (0.5)	3 (2.0)	.081
Other malignancy	11 (2.6)	13 (8.8)	.001
Dyslipidemia	161 (38.2)	54 (36.5)	.719
*Helicobacter pylori* infection (n = 90)‡	27 (47.4)	18 (54.5)	.512

Data are expressed as mean ± standard deviation (SD) or n (%).

BMI = body mass index.

* *P* value for comparing patients with two groups divided by age.

† Others: alcoholic hepatitis, Wilson’s disease, toxic hepatitis, cryptogenic LC

‡ A total of 90 subjects were tested for *Helicobacter pylori* infection. A total of 45 subjects were identified as positive for *Helicobacter pylori* infection.

**Figure. 2.**
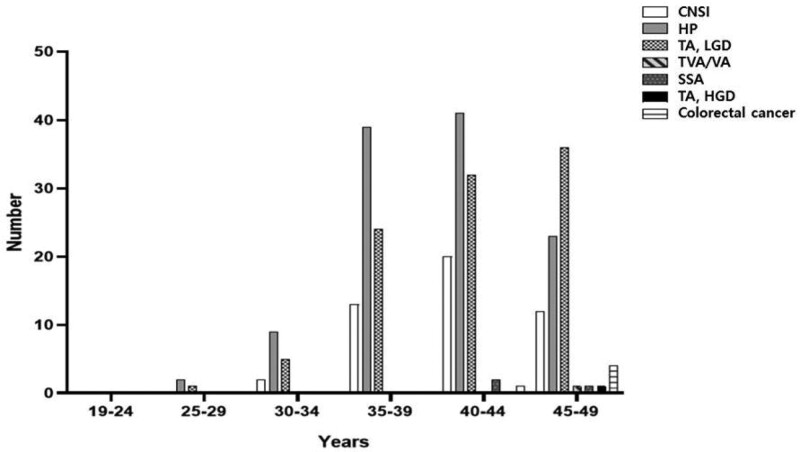
Comparison of histopathological findings by age. HPs (*P* = .027) and tubular adenomas (*P* = .017) show significant differences by age at 5-year intervals. CNSI = chronic nonspecific inflammation, HGD = high-grade dysplasia, HP = hyperplastic polyp, LGD = low-grade dysplasia, SSA = sessile serrated adenoma, TA = tubular adenoma, TVA = tubulovillous adenoma, VA = villous adenoma.

The polyp detection rate was similar between patients aged <45 and ≥45 years (36.7% vs 38.5%; *P* = .699); however, colorectal adenomas <10 mm with low-grade dysplasia were significantly more common in patients aged ≥45 years than in patients aged <45 years (14.9% vs 24.3%; *P* = .009). Furthermore, all polyps over adenomas, except chronic nonspecific inflammations, hyperplastic polyps, were significantly higher in patients aged ≥45 years than in patients aged <45 years (15.4% vs 23.6%; *P* = .023). In addition, the rate of CRC was significantly higher in patients aged ≥45 years than in patients aged <45 years (0.2% vs 2.7%; *P* = .006; Table 4). For detailed differences, we compared the histological results of polyps by dividing the age by 5 years. Hyperplastic polyps and tubular adenomas were significantly different when analyzed at 5-year intervals, and tubular adenomas significantly increased with age (*P* = .017; Fig. 2).

**Table 4:** Characteristics of polyps in 2 groups of subjects divided by age the age of 45.

Variables	19 to 44 y (n = 422, 74.0%)	45 to 49 y (n = 148, 26.0%)	*P* value*
Polyps	155 (36.7)	57 (38.5)	.699
Location			.537
Right colon	70 (45.2)	21 (36.8)	
Left colon	54 (34.8)	22 (38.6)	
Both	31 (20.0)	14 (24.6)	
Morphology			.686
Is	139 (89.7)	48 (84.2)	
Ip	5 (3.2)	3 (5.3)	
Isp	5 (3.2)	2 (3.5)	
IIa	6 (3.9)	4 (7.0)	
Size (mm)	4.59 ± 2.72	7.30 ± 12.22	.103
Number (n)	0.79 ± 1.10	0.94 ± 1.65	.334
Pathologic findings			
CNSI	35 (8.3)	12 (8.1)	.944
HP	90 (21.5)	23 (15.5)	.120
TA, LGD < 10 mm	63 (14.9)	36 (24.3)	.009
TA, LGD ≥ 10 mm	1 (0.2)	1 (0.7)	.437
TVA/VA	0 (0)	1 (0.7)	.091
SSA	2 (0.5)	1 (0.7)	.770
TA, HGD	0 (0)	1 (0.7)	.091
Adenomatous polyps	65 (15.4)	35 (23.6)	.023
Location (n = 100)			.610
Right colon	24 (37.5)	11 (31.4)	
Left colon	24 (37.5)	12 (34.3)	
Both	16 (25.0)	12 (34.3)	
Morphology (n = 99)			.475
Is	59 (92.2)	30 (85.7)	
Ip	1 (1.6)	0 (0)	
Isp	3 (4.7)	3 (8.6)	
IIa	1 (1.6)	2 (5.7)	
Advanced adenomas	3 (0.7)	3 (2.0)	.177
Location (n = 6)			.513
Right colon	2 (66.7)	1 (33.3)	
Left colon	1 (33.3)	1 (33.3)	
Both	0 (0)	1 (33.3)	
Colon cancer	1 (0.2)	4 (2.7)	.006

Data are expressed as mean ± standard deviation (SD) or n (%).

CNSI = chronic nonspecific inflammation, HGD = high-grade dysplasia, HP = hyperplastic polyp, LGD = low-grade dysplasia, SSA = sessile serrated adenoma, TA = tubular adenoma, TVA = tubulovillous adenoma, VA = villous adenoma.

* *P* value for comparing patients with 2 groups divided by age.

### 3.3. Risk factors of colorectal neoplasia

Univariate and multivariable analyses were performed to determine the risk factors for colorectal adenoma and CRC in patients aged <50 years who underwent colonoscopy (Table 5). In univariate logistic regression analysis, age ≥45 years (OR, 1.930; 95% CI, 1.230–3.028; *P* = .004) and colonoscopy due to bowel habit change (OR, 0.339; 95% CI, 0.119–0.971; *P* = .044) were identified as significant factors for colorectal neoplasia. In multivariable analysis, after adjusting for body mass index, fasting glucose levels and presence of hypertension, diabetes, and other malignancies, age over 45 years (adjusted OR, 1.941; 95% CI, 1.187–3.172; *P* = .008) and male sex (adjusted OR, 1.711; CI, 1.044–2.806; *P* = .033) were independent risk factors for colorectal neoplasia.

**Table 5:** Risk factors of colorectal neoplasia.

Variable	Univariate analysis		Multivariable analysis	
	OR (95% CI)	*P* value	Adjusted OR (95% CI)	*P* value
Male sex	1.512 (0.984–2.322)	.059	1.711 (1.044–2.806)	.033
Age over 45 y	1.930 (1.230–3.028)	.004	1.941 (1.187–3.172)	.008
Obesity (BMI ≥ 25 kg/m^2^)	1.090 (0.697–1.703)	.706	0.795 (0.470–1.343)	.391
Fasting glucose	1.003 (0.993–1.013)	.602	1.001 (0.989–1.014)	.842
Cause of colonoscopy				
Screening	1.0 (ref.)		1.0 (ref.)	
Hematochezia, anemia	0.477 (0.164–1.388)	.174	0.466 (0.134–1.620)	.230
Dyspepsia, abdominal pain	0.731 (0.344–1.553)	.415	1.170 (0.512–2.673)	.709
Bowel habit changes	0.339 (0.119–0.971)	.044	0.459 (0.152–1.384)	.166
Weight loss	0.764 (0.088–6.627)	.807	0.488 (0.054–4.395)	.522
Other cancer work up or previous polyp	0.804 (0.266–2.428)	.699	1.088 (0.329–3.594)	.890
Underlying disease				
Hypertension	2.758 (0.979–7.764)	.055	1.874 (0.495–7.091)	.355
Diabetes	1.735 (0.605–4.976)	.306	1.038 (0.271–3.974)	.956
Liver disease	1.021 (0.620–1.679)	.936		
Cardiologic disease	1.108 (0.123–10.017)	.927		
Other malignancy	1.756 (0.714–4.318)	.220	1.242 (0.444–3.478)	.680
Dyslipidemia	1.240 (0.806–1.908)	.328		

BMI = body mass index, CI = confidence interval, OR = odds ratio.

## 4. Discussion

This is the study on the prevalence and risk factors of colorectal adenoma and CRC in a young population aged 19 to 49 years in Korea. Although several studies on the prevalence and risk factors of colorectal adenoma and CRC have been conducted in individuals aged <50 years, most studies were mainly included individuals aged 40 to 49 years. Only a few recent Korean studies have investigated the prevalence and risk factors of colorectal adenoma and CRC in individuals aged 20 to 39 years.^[14,15]^ In our study, the prevalence of colorectal adenoma <10 mm in patients aged 19 to 29, 30 to 39, and 40 to 49 years were 3.2%, 13.8%, and 21.1%, respectively. The prevalence of overall neoplasia and advanced neoplasia, including advanced adenoma and CRC, were 3.2% and 0%, 14.3% and 0.5%, and 24.2% and 3.1%, respectively. These rates were comparable to those reported in some Korean studies or slightly higher than those reported in previous studies.^[14–17,22–24]^ It is possible that the prevalence of advanced neoplasia in our study was higher (3.1%) than that reported in previous studies (2.4%–2.9%) in individuals aged 40 to 49 years in Korea because this study included both asymptomatic and symptomatic participants.^[16,17]^

According to Kang et al,^[25]^ the symptomatic group had a higher prevalence of advanced colorectal adenomas than the screening or surveillance group. However, there was no significant difference in the prevalence of advanced colorectal adenomas between subgroups of the alarm and nonalarm categories (11.7% vs 9.7%, *P* = .056).

In this study, the prevalence of colorectal adenoma and CRC significantly increased in individuals aged 45 to 49 years compared to those aged 19 to 44 years (15.4% vs 23.6%, *P* = .023 and 0.2% vs 2.7%, *P* = .006). In a large-scale Chinese cohort study by Chen et al^[26]^ on colorectal adenoma in the population under the age of 50, the detection rate of colored adenoma increased significantly as the age increased, and in particular, the detection rate increased significantly over the age of 45. In that study by Chen et al, the adenoma detection rate was found to be somewhat lower than in our study, and maybe the reason is that serrated polyp was analyzed separately from adenoma. In the Italian study by Agazzi et al,^[27]^ age as a continuous variable was also associated with the presence of adenomas (incidence rate ratio 1.06; 95% CI, 1.03–1.09; *P* < .001). And, over 40 years of age was a major risk factor for both adenoma and early-onset CRC (OR 2.25; 95% CI, 1.35–3.73; *P* = .002).

Most major guidelines recommend starting screening at the age of 50 years in adults with no family history.^[28–32]^ However, the guideline of the American Cancer Society in 2018 recommended that a screening test be performed according to patient preference at the age of 45 years (qualified recommendation).^[33]^ The evidence for starting screening test at age 45 in the American Cancer Society guidelines is based on several studies.^[34]^ In a recent modeling analysis using large-scale epidemiological data in the United States, when a microsimulated model of a 40-year-old adult was applied in 2015, colonoscopy every 10 years starting at the age of 45 years, sigmoid colonoscopy every 5 years, CT colonoscopy every 5 years, and annual fecal immunochemistry tests were considered effective.^[35]^ In the United States, Canada, Australia, Denmark, New Zealand, and the United Kingdom, the incidence of CRC in individuals aged <50 years, which is not the age required for screening, is increasing. Studies conducted in Asian countries also show an increase in the incidence of CRC in individuals aged <50 years in Taiwan, Japan, and Hong Kong.^[36–40]^ The studies argue that the age at which screening starts should be lowered because of the increase in young CRC patients aged under 50 years. In Korea, the incidence of CRC has been decreasing since 2010 in all age groups, except ≥85 year and 25 to 29 year age groups.^[41]^ In Korea, it is unclear why the incidence of CRC has decreased in recent years in individuals aged <50 years, but compared to other countries, colonoscopy and polypectomy are often performed in Korea even at a young age because access to medical care is relatively good and the cost is inexpensive. Hence, the incidence of CRC appears to have been decreased in individuals aged <50 years, similar to individuals aged ≥50 years.

Several studies on risk factors of CRC have been conducted in the United States. Risk factors for early-onset CRC include genetic factors, male, race, early antibiotic exposure, diabetes, and controllable risk factors such as diet, obesity, smoking, alcohol use, and meat consumption.^[42,43]^ However, just as the difference in CRC incidence varies from country to country, risk factors may not be the same in the United States and Korea because of race, economic status, and region. Nevertheless, it was shown that the risk of CRC was high for those over 45 years of age and men. In a recent large-scale study using data from the National Insurance Corporation in Korea, the age for appropriate CRC screening using Youden index was studied, and an age of 45 years was considered the most appropriate.^[44]^ By lowering the starting age of CRC screening test, it is possible to reduce the incidence and mortality of CRC in young adults, thereby improving the quality of life and reducing social costs. In addition, the present intervention has an effect on increasing the screening rate of CRC in adults aged >50 years. However, if the age for screening test is lowered to 45 years, the screening test rate of individuals at a high risk for colon cancer can be lowered due to the limited number of colonoscopies; and, there is a problem of increasing medical expenses due to the increase in the total number of subjects who underwent colonoscopy. Another major problem is that there is no direct clinical study on the effect of starting colon cancer screening at the age of 45 years. Therefore, when additional analysis were performed on 50- to 55-year-old patients who underwent colonoscopy to compare with 45- to 50-year-old patients (data not shown), it showed that the polyp incidence was significantly increased at the age of 50 to 55 (39, 26.4% vs 81, 67.5%; *P* < .001), but the CRC incidence showed similar rates (4, 2.7% vs 3, 1.9%; *P* = .614). This shows that the occurrence of CRC at the age of 45 and 50 cannot be ignored. Although other risk factors were not compared, further studies are needed.

Our study has several limitations. First, this was a retrospective study in a single-center hospital; hence, selection bias may have occurred. In addition, the number of subjects enrolled in this retrospective study was small and the period of subject selection was short, resulting in selection bias. Previous large-scale studies have shown that male sex, smoking status, alcohol consumption, obesity, abdominal obesity, and metabolic syndrome are risk factors for the development of colorectal neoplasia in young adults aged <50 years.^[14–17]^ However, in our study, age over 45 years old and male sex were significant risk factors of colorectal neoplasia. In addition to the medical records of asymptomatic participants who underwent colonoscopy at the Health Promotion Center, those of symptomatic participants who underwent colonoscopy through the department of gastroenterology were reviewed retrospectively, and there were limitations on the investigation of accurate previous medical history, family history of CRC and social history such as smoking status and alcohol consumption. In order to evaluate whether it is appropriate to start CRC screening at the age of 45, it is necessary to compare the prevalence of colorectal neoplasia between the group below and over the age of 45. Although comparison with the age group of 50 to 55 years, there is a limit to obtaining an accurate cutoff value for the occurrence of CRC, so additional research is needed.

Several studies have reported an association between *H. pylori* infection and colorectal neoplasia,^[45–47]^ but in our study, only 90 patients were tested for the presence of *H. pylori* infection. No statistical analysis was conducted on the correlation between *H. pylori* infection and colorectal neoplasia. To date, there have been no studies in Korea that have examined the association between *H. pylori* infection and colorectal neoplasia in adults aged <50 years. In our study, the prevalence of colorectal adenoma in patients diagnosed with *H. pylori* infection was 24.4%.

## 5. Conclusion

Colorectal adenoma and CRC may occur in young adults aged under 50 years, especially in those aged over 45 years. Based on the results of previous studies and clinical studies to be implemented in the future, of CRC screening from the age of 45 years, we suggest lowering the age at which screening is conducted.

## Author contributions

Guarantor of the article: Y.E.P.

Acquisition of data; analysis and interpretation of data; drafting of the manuscript: S.J.J.

Acquisition of data; study concept and design: J.L.

Study concept and design; critical revision of the manuscript for important intellectual content: E.K., J.S.H., J.L., J.H.C., N.-Y.H., J.P., S.H.P., T.O.K.

Acquisition of data; study concept and design; critical revision of the manuscript for important intellectual content: Y.E.P.

All authors approved the final version of the article, including the authorship list.
